# Multi-response optimization of transesterification parameters of mahogany seed oil using grey relational analysis in Taguchi method for quenching application

**DOI:** 10.1016/j.heliyon.2019.e02167

**Published:** 2019-08-29

**Authors:** R.M. Dodo, T. Ause, E.T. Dauda, U. Shehu, A.P.I. Popoola

**Affiliations:** a Department of Metallurgical & Materials Engineering, Ahmadu Bello University, Zaria, Nigeria; b Department of Chemical, Metallurgical and Materials Engineering, Tshwane University of Technology, Pretoria, South Africa

**Keywords:** Chemical engineering, Industrial engineering, Mechanical engineering, Metallurgical engineering, Petroleum engineering, Materials science, Ester yield, Heat transfer coefficient, Taguchi, Grey relational analysis, Methanol, Catalyst, Temperature, Stirring

## Abstract

This study investigates the possibility of multi-objective optimization in the transesterification of mahogany seed oil (MSO). The Taguchi method together with Grey relational analysis (GRA) was used to maximize both percent fatty acid methyl ester (FAME) yield and heat transfer coefficient (HTC). It was found that methanol to oil molar ratio was the factor that contributed the most in obtaining high percent FAME (ester) yield and HTC. Employing the following conditions: 32.6 wt% methanol (9:1 methanol to oil molar ratio), 0.5 wt% catalyst, 60 °C temperature and 300 rpm agitation was found to offer an improved percent ester yield and HTC. A confirmatory test resulted in an ester yield of 90.1 % and HTC up to 153.0 W/m^2^K. The structure of the optimized transesterified mahogany seed oil (TM) was confirmed by FTIR analysis. In the event of comparison, TM, raw mahogany seed oil (FM) and SAE40 were analyzed by cooling curve analyses. TM identified to have superior quenching performance.

## 1 Introduction

Vegetable and animal oils as a class of fluids have been used for hundreds of years, if not longer, as quenchants for hardening steel. However, when petroleum oils came into being in the early 1900s, the use of these fluids as quenchants became unpopular (Otero et al., 2014). This was primarily due to the fact that generally, they exhibit very poor thermal-oxidative stability and low coefficient of heat transfer at high temperatures (Ramesh and Prabhu, 2014). There has been renewed interest in the use of vegetable oils as quenchant (bioquenchant) due to its less polluting and renewable nature as against the conventional petroleum-derived quenching oils (Ghadge and Raheman, 2006). However, the relatively poor thermal-oxidative stability has continued to be a challenge for their wide usage in heat treatment industries (Fernandes and Prabhu, 2008). Modification of the molecular structure of the vegetable oils certainly overcomes these limitations. Adjusting the chemical structure of the vegetable oils can be attained in many ways, including; anti-oxidants additions, genetic and chemical modifications. Transesterification is one of the processes of chemical modification. It is a process whereby vegetable oil/fat is chemically reacted with an alcohol in the presence of a catalyst (NaOH or KOH) to form glycerol and ester. If methanol is used in the process, the ester produced is called fatty acid methyl ester (FAME) (Knothe, 2001).

Chemically, vegetable oils and animal fats are high molecular esters of fatty acids. These are known as triglycerides of fatty acids (TG). These TG in general, have the molecular weight in the order of 800 g/mol or more (Mahesh Babu et al., 2018). Due to their large weights; these oils have high viscosity causing limitations in their exploitation as quenchants. Fernandes and Prabhu (2008) reported that cooling rate offered by quenching oils is strongly dependent on the viscosity of the oils. Oils with higher viscosity offer greater resistance to the motion of vapour bubbles during the nucleate boiling stage and the supply of cold liquid to the heated surface is reduced. Thus, this results in a lower heat transfer coefficient (HTC) which is a consequence of poor heat transfer in the quenchant during quenching operation. Through transesterification, these TG molecules are broken apart and reformed into simpler molecules (monoesters) which are lighter and less viscous (Ashok et al., 2014). Further, the transesterification leads to a high conversion of triglycerides (TG) into methyl esters (Marjanović et al., 2010) with diglycerides (DG) and monoglycerides (MG) as reaction intermediates and glycerol as a by-product. Indeed, the overall process is a sequence of three consecutive steps, which are reversible reactions. In the first step, diglycerides (DG) are obtained from triglycerides (TG); monoglycerides (MG) are then produced from diglycerides and in the last step, glycerol (G) is obtained from monoglycerides (Richard et al., 2013). In all these reactions, methyl esters are produced. The viscosity of the transesterified oil (TO) is related to the presence of the reaction intermediates. A high content of intermediates resulted to high viscosity which leads to lower HTC. On this account, experimental conditions of the transesterification reaction have a significant influence on the HTC of the TO. Similarly, according to literatures (Bruno et al., 2006; Rashid and Anwar, 2008; Sharma et al., 2008), transesterification parameters such as molar ratio of vegetable oil to alcohol, catalyst type and amount, reaction and temperature and the degree of agitation have profound impact on the percent ester yield after transesterification.

The transesterification process as highlighted involves many parameters that affect the reaction and optimizing these parameters requires a large number of experiments, which is laborious, time-consuming and economically non-viable. The Taguchi method has been widely applied in manufacturing to robustly design a product or process with a single quality characteristic (Pan et al., 2007).

The change of heat flux density during quenching operations is associated with the temperature difference between the hot surface of the component and the liquid (Hasan et al., 2018). The transfer of heat as the consequence of the temperature difference is normally quantified in terms of the heat transfer coefficient. Thus, the heat transfer coefficient of transesterified bioquenchants is as important as its percent ester yield. Hence, maximizing the two quality characteristics simultaneously will make the whole process cost-effective. In the optimization of multiple response characteristics, Taguchi based grey relational analysis (GRA) can be employed. Designing experiments through the method has been used quite successfully in multi-response optimization by Sylviana et al. (2015) and Ilo et al. (2012).

The present study focuses on the optimization of the process parameters for maximum percent ester yield and HTC in transesterification of FM using Taguchi based GRA approach.

## 2 Materials and methods

### 2.1 Transesterification process of the esterified oil

After the esterification reaction where the percent free fatty acid (%FFA) was reduced to a value of less than 0.5%, transesterification was carried out. Multi-response optimization analysis of FAME yield and HTC with Taguchi-based GRA was conducted. The optimization analyses were carried out according to the criteria the-larger-the-better. The Taguchi method with an L9 orthogonal array was implemented (Table 1). Four experimental factors were used namely menthol to oil molar ration (amount of methanol), amount of catalyst (catalyst loading), temperature and stirring speed (agitation).

**Table 1:** DOE for transesterification process of MSO.

Expt.No.	Factors			
	Methanol/Oil ratio (wt %)	Catalyst (wt %)	Temperature (°C)	Agitation (rpm)
1	10.9	0.5	40	300
2	10.9	1.0	50	450
3	10.9	1.5	60	600
4	21.7	0.5	50	600
5	21.7	1.0	60	300
6	21.7	1.5	40	450
7	32.6	0.5	60	450
8	32.6	1.0	40	600
9	32.6	1.5	50	300

For 100g of oil sample, 3:1, 6:1 and 9:1 methanol to oil molar ratios were equivalent to 10.9, 21.7 and 32.6wt% of methanol respectively. The optimal experimental conditions were obtained from the design of experiment (DOE) using MINITAB 16 statistical software.

The transesterification process during optimization was carried out as follows:

The calculated mass of the NaOH was dissolved into the measured methanol and the mixture was then poured into 100g of the oil sample. Next, the mixture was placed on a magnetic stirrer at 60 °C for stirring and heated for 1 h. Finally, the solution was poured into a separating funnel for gravitational settling for the separation of FAME and glycerol. The denser glycerol was drained. Afterwards, the ester was purified by water washing and then drained.

Results of the transesterification reaction were evaluated in terms of percent FAME yield (ester yield) and heat transfer coefficient (HTC). The percent ester yield was calculated using Eq. (1).

(1) $$\text{\%}\;\text{ester}\;\text{yield}=\frac{\text{Total}\;\text{weight}\;\text{of}\;\text{methyl}\;\text{esters}\;\;}{\text{Total}\;\text{weight}\;\text{of}\;\text{oil}\;\text{and}\;\text{methanol}\;\;}\;\;\text{×}\;100$$

**Fig. 1:**
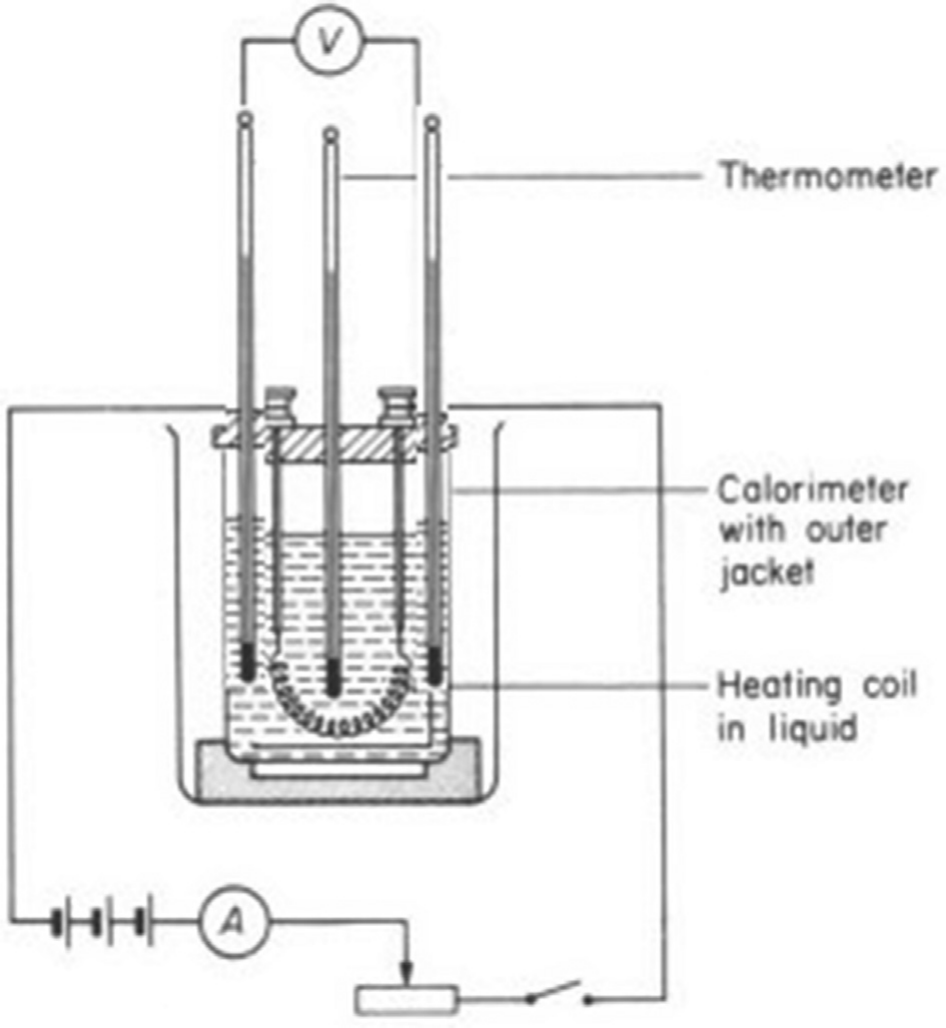
Experimental set up for the determination of optimized HTC.

HTC was determined using the following method:

A heating coil was fixed at one of the interior sides of a well-lagged square based rectangular calorimeter. Three thermometers were fixed directly opposite to the coil at three different locations at the other interior side. The coil was connected in series with a D.C. supply, an ammeter, a rheostat, and a plug key or switch. A voltmeter was connected directly across the coil terminals (Fig. 1). A preliminary test was carried out for the purpose of adjusting the current to the value that would give a temperature rise of about 10 °C. The current was then switched on. After a temperature rise of about 10 °C, it was then switched off. Voltmeter reading was noted. Heat dissipated and the associated heat transfer coefficient was calculated using the following basic equations.

(2) Q=VI

(3) $$T_{i}=\frac{T_{1}+T_{2}+T_{3}}{3}$$

(4) $$h=\;\frac{Q}{{A\left(T_{i}-T_{r}\right)}_{\;}}$$

Where, Q = Heat generated per unit second by the heating coil, WV = Voltmeter reading, VI = Ammeter reading, AT_1_, T_2_, T_3_ = Temperature readings of thermometers, °CT_i_ = Average temperature of the oil sample at the other interior side, °Ch = Heat transfer coefficient of oil sample, W/m^2^KA = Total surface area of the rectangular calorimeter, m^2^, (*SA* = *hs*+*s*^2^)Tr = Room temperature, °C

The FTIR spectra were recorded on CARY 630 FTIR model of Agilent technologies in a scanning range of 650–4000 cm-^1^ for 16 scans at a spectral resolution of 8 cm-^1^. A very thin film of KBr pellet was prepared to introduce the sample for the spectral analysis. The spectra were recorded in transmittance mode. On the other hand, Cooling curves were developed under non-agitated conditions according to ASTM D6200–01 methods. Further, the temperature dependent heat flux values and the heat transfer coefficient of the oils were calculated from measured cooling curve data using the following Equation (Goryushin et al., 1999)

(5) $$\text{q}=\text{h}\;\left({\text{T}}_{\text{p}}-{\text{T}}_{\text{q}}\right)=\;\frac{{\text{c}}_{\text{p}}}{\text{A}}\rho V\;\frac{{\text{dT}}_{\text{p}}}{\text{dt}}$$

where q is the heat flux value of the quenchant, h is the heat transfer coefficient between the probe surface and oil sample, A is the surface area of the probe that surrounds the oil sample, Tp is the probe temperature, Tq is the quenchant temperature (oil sample), c_p_ is the specific heat of the probe sample, ρ is the density of the probe sample, V is the volume of the probe sample, t is the time and (dT_p_)/dt is the cooling rate of the oil sample.

### 2.2 Grey relational generation

In the generation, the first step is the normalization of the signal-to-noise (S/N) ratio values in the range between 0 and 1. The grey relational coefficient (GRC) is then calculated from the normalized S/N ratio to indicate the relationship between the desired and the actual S/N ratio. In the subsequent step, the grey relational grade (GRG) is calculated by taking the mean of the grey relational coefficient corresponding to each response. The grey relational grade is used for the overall evaluation of the process.

The present investigation targeted on the optimization of percent ester yield and HTC. So on this account, the normalization of the S/N ratios was carried out according to the criterion of the performance characteristics the-larger-the-better. The normalized S/N ratio X_ij_ for the ith performance characteristic in the jth experiment was calculated using Eq. (6) (Sudeepan et al.*,* 2014) and the results are presented in Table 2.

(6) $$X_{ij}=\;\frac{Y_{ij}-\text{min}\left(Y_{ij}\right)}{\text{max}\left(Y_{ij}\right)-\text{min}\left(Y_{ij}\right)}$$

Where: X_ij_ is the normalized S/N ratioY_ij_ is the S/N ratio obtained from Taguchi analysismax (Y_ij_) and min (Y_ij_) are respectively the maximum and minimum values of S/N ratios

**Table 2:** Normalized S/N ratios for transesterification.

Exp. No.	% Ester yield	% Ester yield S/N ratio (dB)	HTC, *h* (W/m^2^K)	HTC S/N ratio (dB)	Normalized S/N ratio Data	
					(% Ester yield)	(HTC)
1	78.27	37.87191	134.485	42.57348	0.467706	0.042818
2	78.01	37.84301	139.717	42.90499	0.454891	0.332709
3	69.32	36.81717	133.729	42.52451	0.000000	0.000000
4	89.87	39.07229	142.397	43.07002	1.000000	0.477023
5	86.06	38.69603	152.547	43.66807	0.833150	1.000000
6	87.86	38.87582	144.691	43.20883	0.912878	0.598410
7	89.81	39.06649	151.797	43.62526	0.997428	0.962565
8	88.7	38.95847	147.547	43.37861	0.949527	0.746873
9	87.09	38.79937	152.091	43.64207	0.878974	0.977261

In the subsequent step, deviation sequence (Δ) and the GRC were calculated from the normalized values according to Eqs. (7) and (8) respectively (Mahesh Babu et al., 2018). In the study, equal weight was given to the responses where the distinguishing coefficient ζ equal to 0.5 was used in the calculation. This value is widely adopted by many researchers (Sylviana et al., 2015; Mahesh Babu et al., 2018). Table 3 depicts the deviation sequence (Δ) and the GRC for the transesterification. Afterwards, the GRG was calculated by Eq. (9) (Pan et al. (2007) and given in Table 4.

(7) $$\Delta_{0j}\left(k\right)=\;||X_{0}\left(k\right)-\;X_{j}\left(k\right)||$$

(8) $$GRC_{j}\left(k\right)=\;\frac{\Delta_{min}+\;\zeta \Delta_{max}}{\Delta_{0j}\left(k\right)+\zeta \Delta_{max}\;}$$

$$0<GRC_{j}\left(k\right)\leq 1$$

Where:j = 1,2,…n; k = 1, 2,….m, n is the number of experimental data items and m is the number of responses.$X_{0}\left(k\right)$ is the reference sequence (*X*_*o*_ *(k)* = 1, k = 1, 2…m);*X*_*j*_*(k)* is the normalized value for *a j*th experiment for the *k*th performance characteristics$\Delta_{0j}$ is the deviation sequence, which is an absolute value of the difference between the ideal sequence and the normalized sequence$\Delta_{max}$ and $\Delta_{min}$ are respectively the minimum and maximum values of. $\Delta_{0j}$ζ is the distinguishing coefficient, which is defined in the range 0≤ζ≤1, If equal weight is given to the responses like in the present study, then ζ is 0.5.

(9) $$GRG_{j}=\;\frac{1}{m}\;\overset{m}{\underset{k=1}{\sum }}GRC_{j}\left(k\right)$$

**Table 3:** Deviation sequence and GRC.

Exp. No.	Deviation sequence (Δ)		GRC	
	Ester yield	HTC	Ester yield	HTC
1	0.532294	0.957182	0.484358	0.343128
2	0.545109	0.667291	0.478419	0.428342
3	1.000000	1.000000	0.333333	0.333333
4	0.000000	0.522977	1.000000	0.488769
5	0.166850	0.000000	0.749794	1.000000
6	0.087122	0.401590	0.851612	0.554576
7	0.002572	0.037435	0.994882	0.930344
8	0.050473	0.253127	0.908310	0.663899
9	0.121026	0.022739	0.805119	0.956501

**Table 4:** Grey relational grade and its order.

Exp. No.	Grey relational grade (GRG)		
	GRG	S/N ratio	Order
1	0.413743	-7.66538	8
2	0.453380	-6.87074	7
3	0.333333	-9.54243	9
4	0.744385	-2.56405	5
5	0.874897	-1.16086	3
6	0.703094	-3.05974	6
7	0.962613	-0.33097	1
8	0.786105	-2.09039	4
9	0.880810	-1.10236	2

The multi-objective characteristics could be analyzed using the grade values listed in Table 4. The grey relational grade and their orders are presented in the Tables. According to the grey relational analysis, the experimental conditions of any experiment with the maximum grade value will be the one near the optimum condition. Therefore, from Table 4, it can be observed that control parameters’ setting of experiment 7 had the greatest GRG and this indicates that experiment 7 is the near optimal transesterification factors setting for maximum Ester yield and HTC simultaneously among the chosen nine experiments. According to the GRG order, experiment 9 and 3 valued second best and least respectively. The overall mean of the grey relational grade was obtained to be 0.6836.

## 3 Results and discussion

### 3.1 Optimization results

#### 3.1.1 Effect of each transesterification process parameter on the GRG

The influence of each transesterification process parameter on the grey relational grade at different levels can be identified since the experimental design employed was orthogonal. Therefore, the mean for each process parameter of each level has been found through the Taguchi method. Using MINITAB 16 statistical software, the experimental data for GRG were converted into S/N ratio according to the larger-the-better criterion. The mean of the GRG for each level of the transesterification process parameters is summarized and presented in Table 5. The order of influence of the factors is demonstrated by the ranking which is based on the delta values. The delta value is the difference between the minimum and the maximum value of the average GRG of each process parameter. Methanol to oil molar ratio with the highest delta value of 0.4764 was found to have the most substantial effect, among the various transesterification process parameters, for maximizing ester yield and HTC (Table 5). The other control factors have a less significant effect. The order of their importance is agitation, temperature and amount of catalyst.

**Table 5:** Response table for GRG.

Level	Methanol	Catalyst	Temperature	Agitation
1	0.4002	0.7069	0.6343	0.7231
2	0.7741	0.7048	0.6929	0.7064
3	0.8765	0.6391	0.7236	0.6213
Delta	0.4764	0.0678	0.0893	0.1019
Rank	1	4	3	2

**Fig. 2:**
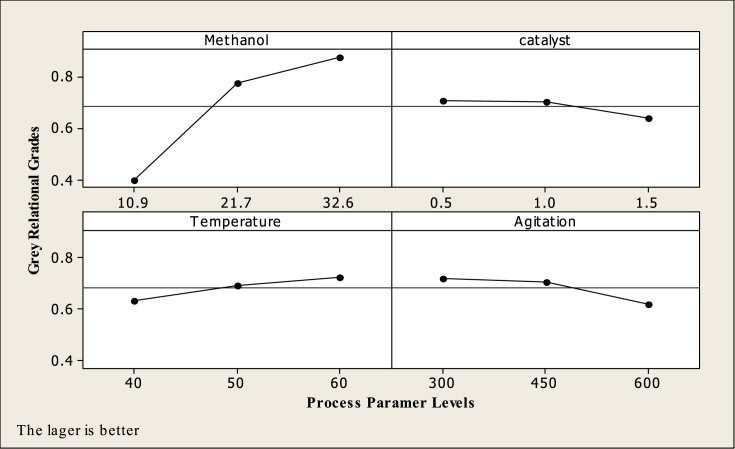
Influence of process parameters on GRG.

Fig. 2 illustrates the grey relational grade plot, where the middle-horizontal line in Fig. is the value of the total mean of the GRG. The point near to the horizontal line has a less significant effect and the one which has the highest inclination will have the most striking effect on the two quality characteristics (ester yield and HTC). It is clear from the Fig. that methanol to oil molar ratio with the highest inclination at level 3 had the most powerful effect on the responses. Agitation is the second most influential factor with the highest point at level 1. Next is temperature followed by catalyst with the highest inclination at level 1. Consequently, the GRG increased with the amount of methanol for the transesterification of the oil. The influence of the amount of methanol on the GRG is attributable to the fact that the transesterification reaction is a reversible reaction. Thus, excess methanol leads to more ester yield. Similarly, as the giant molecules of triglycerides convert into smaller methyl esters, the viscosity decreases, therefore the transfer of heat becomes more rapid. This justified the increase in HTC with respect to GRG. The results are alike with the previous study (Rajan and Rajesh, 2017). Additionally, Fig. 2 depicted the peak value of each process parameters namely 32.6 wt% of methanol, 0.5 wt% of catalyst, 60 °C of temperature and 300 rpm of agitation.

#### 3.1.2 Result of the confirmation experiment

The confirmation test was performed in order to validate the experimental results. The predicted grey relational grade $\bar{\gamma }$ at the optimized level of the process parameters were calculated using the model Eq. (10) (Sudeepan et al., 2014).

(10) $$\bar{\gamma }=\;\gamma_{m}+\;\overset{q}{\underset{i=1}{\sum }}\left(\bar{\gamma_{i}}-\;\gamma_{m}\right)$$

Where: $\gamma_{m}$ is the overall mean GRG${\bar{\gamma }}_{i}$ is the mean GRG at the optimal level of the *i*th parameterq is the number of design parameters

The comparisons of the predicted and the experimental grey relational grade are shown in Table 6. It is seen that the optimal parameter combination improves the grey relational grade from 0.9626 to 0.9687 by 0.892 %. Likewise, the percent ester yield and HTC increased from 89.81 to 90.1 % and 151.797–153.001 W/m^2^K respectively. The prediction percent errors calculated with the experimental data as reference was found to be 1.108 %. Therefore, the results of the confirmation experiments supported the accuracy and the validity of the model Eq. (10) for predicting optimum per cent ester yield and HTC in the present study, since the percent errors obtained are within the 5% confidence interval of the predicted optimum condition as obtained by Jayaraman and Kumar (2014).

**Table 6:** Confirmation test for GRG.

	Initial best process parameter (Exp. No. 7)	Optimal process parameter		Improvement		Prediction Error
		Prediction	Experiment			
Level	M_3_C_1_T_3_A_2_	M_3_C_1_T_3_A_1_	M_3_C_1_T_3_A_1_	Value	%	%
GRG	0.96261	0.97940	0.96867	0.0061	0.892	1.108
% Ester yield	89.810	-	90.102			
HTC (W/m^2^K)	151.797	-	153.001			

### 3.2 Characterization methods

The transesterified mahogany seed oil (TM) produced using the optimum parameters setting was subjected to FTIR and cooling curve analyses. In addition, heat transfer coefficient and heat flux density at high temperatures were estimated from the cooling curves. The same analyses were run on the raw mahogany seed oil (FM) for comparison. The results are presented in the following sections.

#### 3.2.1 Fourier transformed infrared spectroscopy (FTIR)

**Fig. 3:**
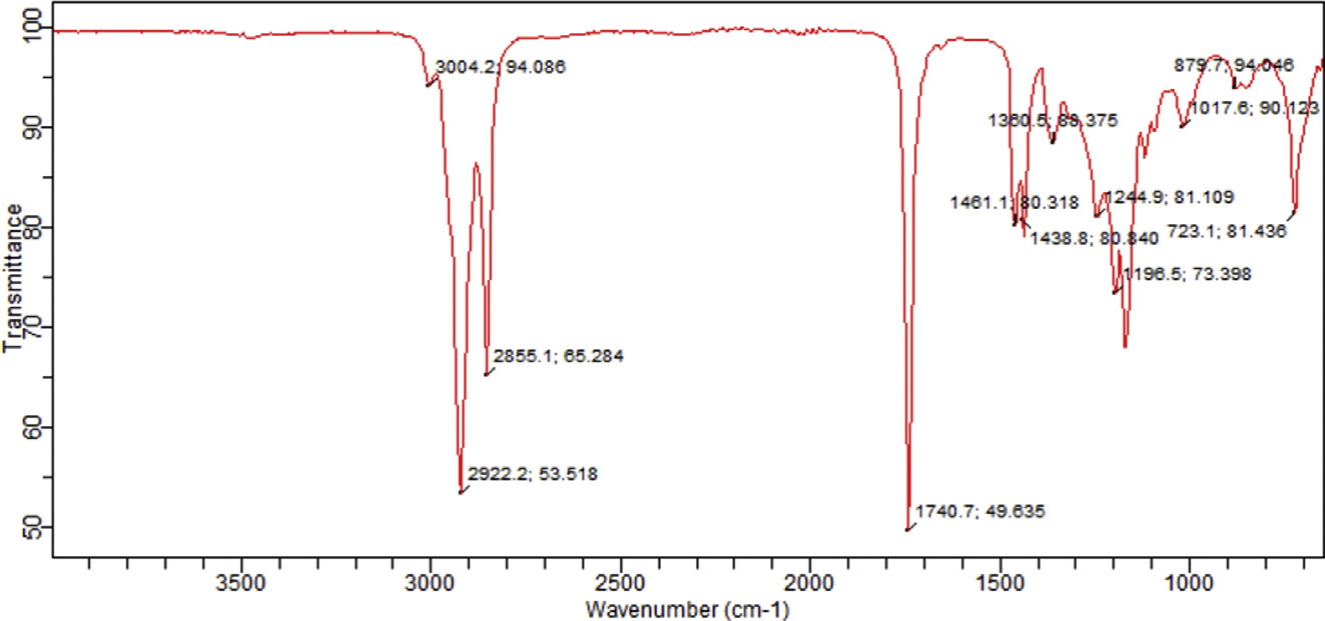
FT-IR spectrum of TM.

**Fig. 4:**
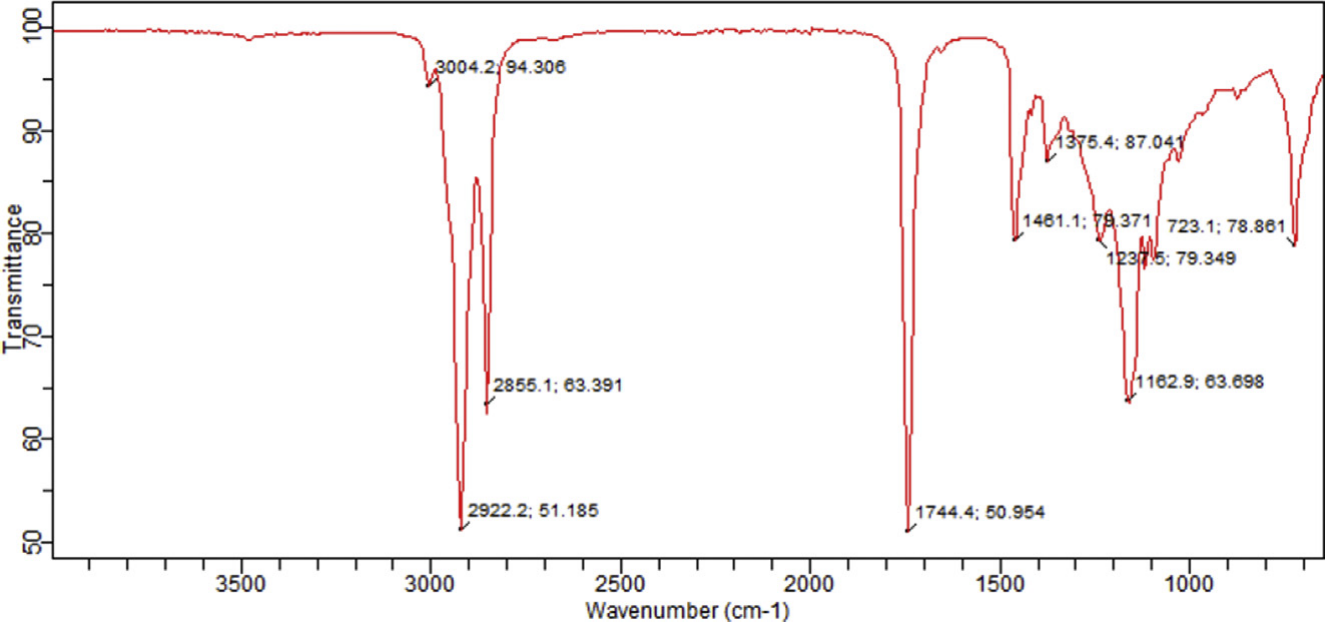
FT-IR spectrum of FM.

**Fig. 5:**
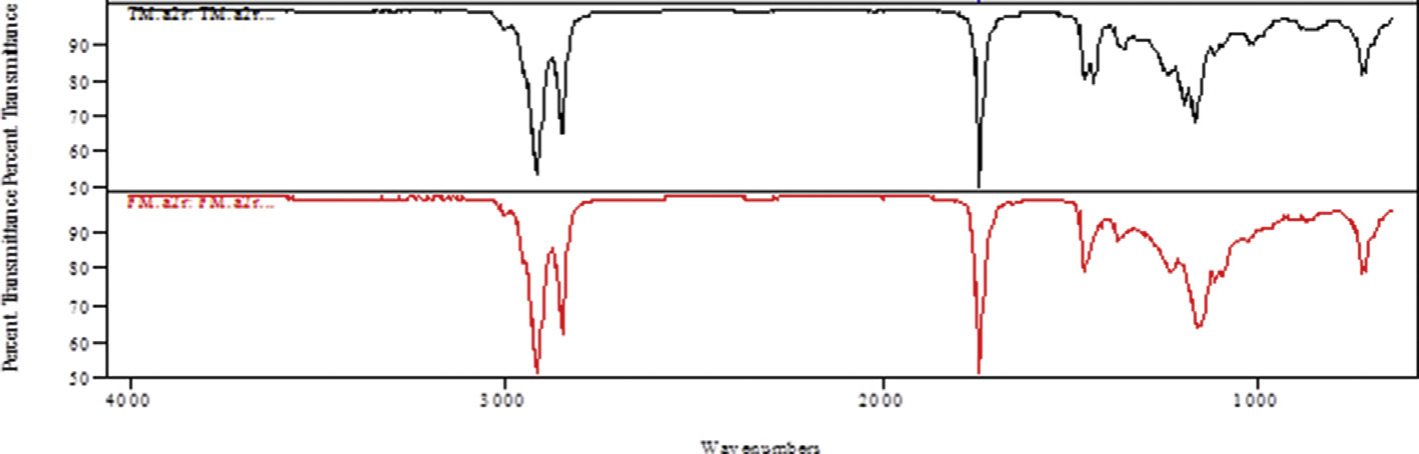
Stacked FT-IR spectra of raw and transesterified MSO.

The spectra from the FT-IR analysis for the raw and transesterified bioquenchants displayed several absorption peaks as illustrated in Figs. 3, 4, and 5. In the spectra, characteristic peaks at 3474 cm^−1^, 3004.2 cm^−1^ and 1653 cm^−1^ were observed which correspond to an overtone stretching vibration of C=O for the esters, vibrational stretching of C–H in C=C–H and unsaturation in the fatty acids present respectively. Conversely, the presence of CH_3_- methyl ester group in the TM is indicated at wavenumber 1438.8 cm^−1^. This is alike with the observation of Siatis et al. (2006).

#### 3.2.2 Cooling curve analysis

**Fig. 6:**
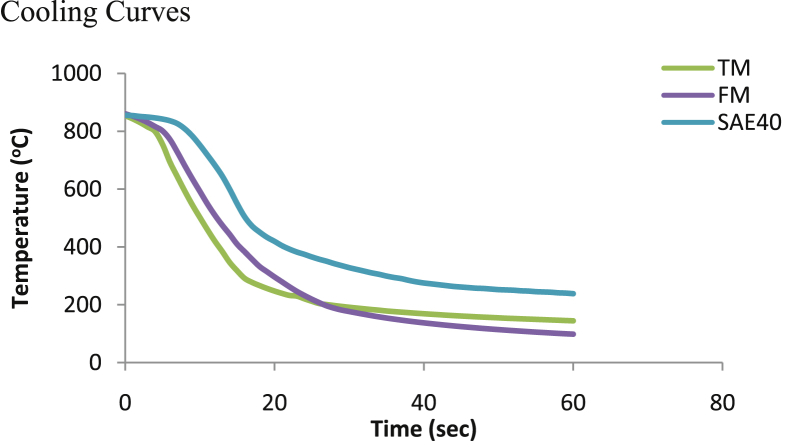
Cooling curves of the transesterified and raw MSO using the INCONEL 600 probe.

Fig. 6 indicates the developed cooling curves for the modified and unmodified bioquenchants. TM proved to have the shortest stage I (vapor blanket stage); which was estimated to last for 3 s, whereas stage II was observed to last for 13 s. Conversely, vapor blanket ruptured at a longer time in FM. Accordingly, the data suggest that chemical modification on the bioquenchants substantially accelerate vapor blanket rupture. In a similar occurrence, Simêncio, et al. (2016) recorded a shorter vapor blanket in palm oil due to improved thermal-oxidative stability as a consequence of antioxidant addition. SAE40 mineral oil presented a prolonged vapor blanket and boiling stage enabling it to have slower cooling than TM and FM. This is in line with what was established by Komatsu et al. (2009).

**Fig. 7:**
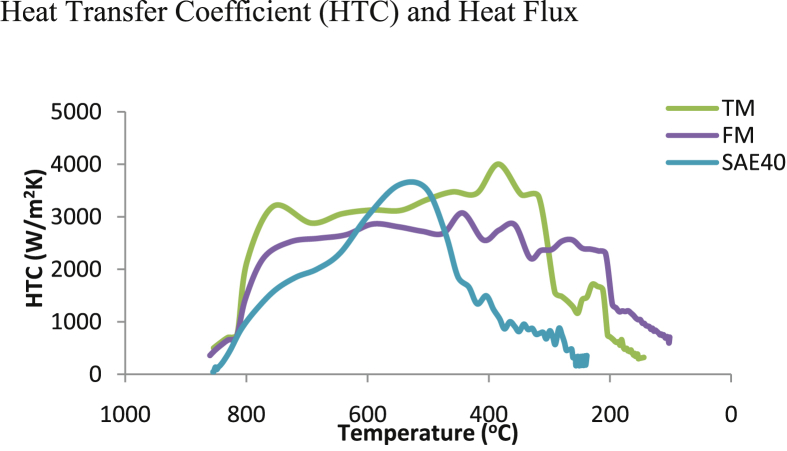
HTC as a function of surface temperature for transesterified and raw MSO.

The dependence of HTC on the surface temperature during quenching operation is presented in Fig. 7 for transesterified and raw bioquenchants. In TM and FM, HTC increases markedly after a minor rise at few temperature drops on immersion. TM gave the highest HTC at 384 °C. Next to it is SAE40 with HTC of 3548 kW/m^2^K at 504 °C. However, amongst all the quenchants, FM exhibited the least peak value of HTC. The outstanding HTC displayed by TC could be connected to the molecular structure modified after transesterification. HTC decreases after reaching the peak values in a fluctuating manner in all the quenchants. This is similar to the results of Buczek and Telejko (2013).

**Fig. 8:**
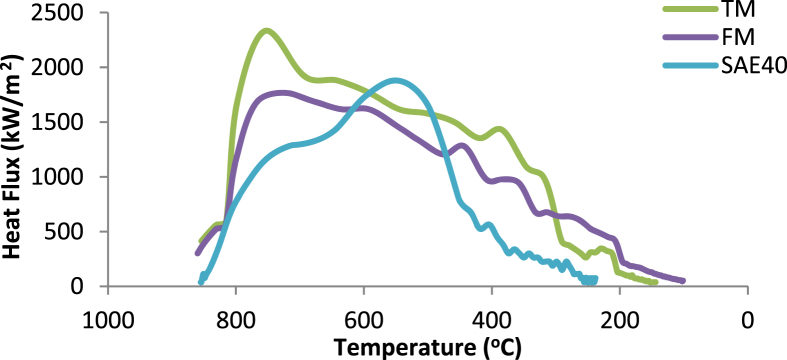
Heat Flux values as a function of surface temperature for transesterified and raw MSO.

Fig. 8 demonstrates plots of the heat flux values versus surface temperatures for transesterified and raw MSO. Immediately on immersion, flux values were low due to the slow cooling effect of the stage I. Additionally, TM offers the highest maximum heat flux value followed by SAE40 and then FM. Alike result was reported by Durowoju et al. (2013).

## 4 Conclusions

In the present investigation, optimization in percent ester yield and HTC in the transesterification of the MSO was successfully carried out by using the Taguchi-Grey relational analysis approach. The experimental factors considered are methanol to oil molar ratio, catalyst concentration, temperature and agitation (stirring speed). The analysis indicated that input parameters such as methanol to oil molar ratio and agitation showed a powerful effect on the percent ester yield and HTC. The conclusions of the study are given as follows:

➢ The optimum values of input variables influencing the transesterification process are 9:1 methanol-to-oil molar ratio (32.6 wt% of methanol), 0.5 wt % catalysts loading, 60 °C temperature and 300 rpm agitation.
➢ The methanol-to-oil molar ratio is the most substantial process parameter affecting the percent ester yield and HTC whereas catalyst concentration is the least significant parameter.
➢ The results of the confirmation experiments with GRG of 0.9687, assured the accuracy and reliability of the GRG model Equation developed for predicting optimum percent ester yield and HTC.
➢ FT-IR analysis confirmed the formation of methyl esters in the TM produced using the optimum parameters setting.
➢ From the cooling curve analysis; the TM obtained using the optimum experimental variables displays splendid HTC. Hence, amongst the tested quenchants, TM has shown superior quenching performance.

## Declarations

### Author contribution statement

R. M. Dodo: Conceived and designed the experiments; Performed the experiments; Wrote the paper.

T. Ause, E. T. Dauda: Analyzed and interpreted the data.

U. Shehu, A. P. I. Popoola: Contributed reagents, materials, analysis tools or data.

### Funding statement

This work was supported by the Management of Ahmadu Bello University, Zaria, Nigeria through the Tertiary Education Trust Fund (TETFund).

### Competing interest statement

The authors declare no conflict of interest.

### Additional information

No additional information is available for this paper.
